# Outcome of Non-small Cell Lung Cancer Patients With N3 Stage: Survival Analysis of Propensity Score Matching With a Validated Predictive Nomogram

**DOI:** 10.3389/fsurg.2021.666332

**Published:** 2021-04-30

**Authors:** Chang Han, Yijun Wu, Xu Sun, Yuming Chong, Kai Kang, Zhikai Liu, Fuquan Zhang

**Affiliations:** ^1^Department of Radiation Oncology, Peking Union Medical College Hospital, Chinese Academy of Medical Sciences & Peking Union Medical College, Beijing, China; ^2^Peking Union Medical College, Eight-year MD Program, Chinese Academy of Medical Sciences, Beijing, China; ^3^Department of Endocrinology, Peking Union Medical College Hospital, Chinese Academy of Medical Sciences & Peking Union Medical College, Beijing, China

**Keywords:** non-small cell lung cancer, N3 stage, surgery, radiotherapy, chemotherapy

## Abstract

**Background and Objectives:** N3-positive non-small cell lung cancer (NSCLC) is usually regarded as inoperable. There were very few studies that focused on N3-NSCLC patients. This study aims to analyze prognosis of NSCLC patients with N3 disease and provides retrospective indications.

**Methods:** NSCLC patients staged as N3 were retrospectively reviewed from the Surveillance, Epidemiology, and End Results database. Univariate and multivariate Cox regression were used for identifying prognostic factors. The selected predictive parameters by the least absolute shrinkage and selection operator (LASSO) regression were used to develop predictive nomogram models for overall survival (OS) and lung cancer-specific survival (CSS). The C-index values were calculated to assess the models' predictive ability, while calibration curves were plotted to evaluate the agreement between the predicted and the actual survival. Survival curves were plotted by Kaplan-Meier method and were compared by log-rank test. Propensity score matching (PSM) was used to balance the baseline characteristics between treatment groups.

**Results:** A total of 24,747 N3-NSCLC patients were enrolled. The 1-, 3-, and 5-year OS rates were 35.8, 6.8, and 1.7%, respectively, while the corresponding CSS rates was 36.6, 6.9, and 1.8%, respectively. The nomogram models were developed using 11 significant prognostic parameters, including age, sex, race, histology, stage, T stage, bone, brain and liver metastases, surgery, and chemotherapy. Both of them demonstrated great predictive ability and performed well in the calibration curves. After PSM, patients receiving surgery demonstrated significantly better survival than those who did not. Besides, there was no significant difference of survival between patients receiving chemotherapy with and without radiotherapy.

**Conclusions:** The nomogram models for predicting survival outcome of N3-NSCLC patients can be clinically used. Surgery may be beneficial to the survival for these patients, while radiotherapy may not have additional survival benefits in patients receiving chemotherapy.

## Introduction

Non-small cell lung cancer (NSCLC) patients with N3 stage are usually considered inoperable, while chemoradiation therapy predominates in the following managements (1, 2). According to the newly eighth edition American Joint Committee on Cancer (AJCC) tumor-nodes-metastasis (TNM) staging system (3), positive N3 has been defined as the involvement of scalene zone, the supraclavicular or contralateral hilar/mediastinal lymph nodes, and N3 patients can be staged as IIIB or later. In the previous studies, concurrent chemoradiotherapy has been confirmed to improve prognosis of IIIB patients (1, 2, 4–7). However, very limited number of patients with N3 involvement were included in these studies, especially for those with positive supraclavicular nodes. The heterogeneity of IIIB patients made it more difficult to accurately identify the optimal modalities among various subgroups. There were very few studies that focused on the prognosis and clinical outcome of N3-positive NSCLC patients.

In spite of the previous viewpoint that surgical resection was not recommended for IIIB patients, especially with N3 disease, it is now reconsidered to improve survival outcome combining with induction therapy. The retrospective study of the National Cancer Database from Raman et al. demonstrated that surgical resection improved long-term survival compared with chemoradiotherapy in NSCLC patients with N3 disease (8). As evidences have been rare, the role of surgery in this population requires further discussion. Whether surgery combining with chemoradiotherapy is a benefit to the survival outcome has not been clearly clarified. There was no research that reported the prognostic study or predictive model for N3-positive NSCLC.

In the present study, from the Surveillance, Epidemiology, and End Results (SEER) database, we first identified NSCLC patients with positive N3 to find the associated prognostic factors and the optimal therapeutic strategy. Besides, predictive models for survival outcome of N3 patients were developed and validated. We present the following article in accordance with the STROBE reporting checklist.

## Materials and Methods

### Study Population

Patients diagnosed with N3-positive NSCLC patients between 2010 and 2015 were identified from the nine registries of the SEER database (http://seer.cancer.gov/): Atlanta, Connecticut, Detroit, Hawaii, Iowa, New Mexico, San Francisco–Oakland, Seattle–Puget Sound, and Utah. The database was established by the National Cancer Institute of the United States, which collected and recorded epidemiological data, clinical demographics, therapeutic information, and follow-up status of approximately 30% of cancer patients in the U.S. Nowadays its clinical information is publicly available to clinicians all worldwide. Thus, patients' information is anonymous, and the informed consent is not required.

Based on the database, patients that met the following criteria were included: (1) primary NSCLC (ICD-O-3/WHO 2008, International Classification of Diseases for Oncology, Third Edition); (2) clinical N3 stage (AJCC, 7th edition); (3) age at diagnosis: 18-years or older; and (4) year of diagnosis: 2010–2015. The exclusion criteria were as follows: (1) not the first primary cancer; (2) small cell lung cancer; (3) survival time <1 month; and (4) incomplete staging, treatment, and follow-up information. The latest update of follow-up information was in November 2018.

### Study Variable

Multiple variables related to N3-NSCLC patients were extracted from the SEER database, including age, sex, race [white, black, others (American Indian/AK Native, Asian/Pacific Islander)], diagnosis year (2010–2015), laterality (right, left and unknown), primary site (upper, middle, lower lobe, main bronchus, overlapping lesion, and unknown), stage (IIIB and IV), T stage (T1–T4 and TX), distant metastasis (bone, brain, liver, and lung metastasis), histology (adenocarcinoma, squamous cell carcinoma, and other NSCLC), grade (I–IV and unknown), surgery (yes and no), chemotherapy (yes and no/unknown), and radiotherapy (yes and no/unknown).

### Statistical Analysis

**Figure 1:**
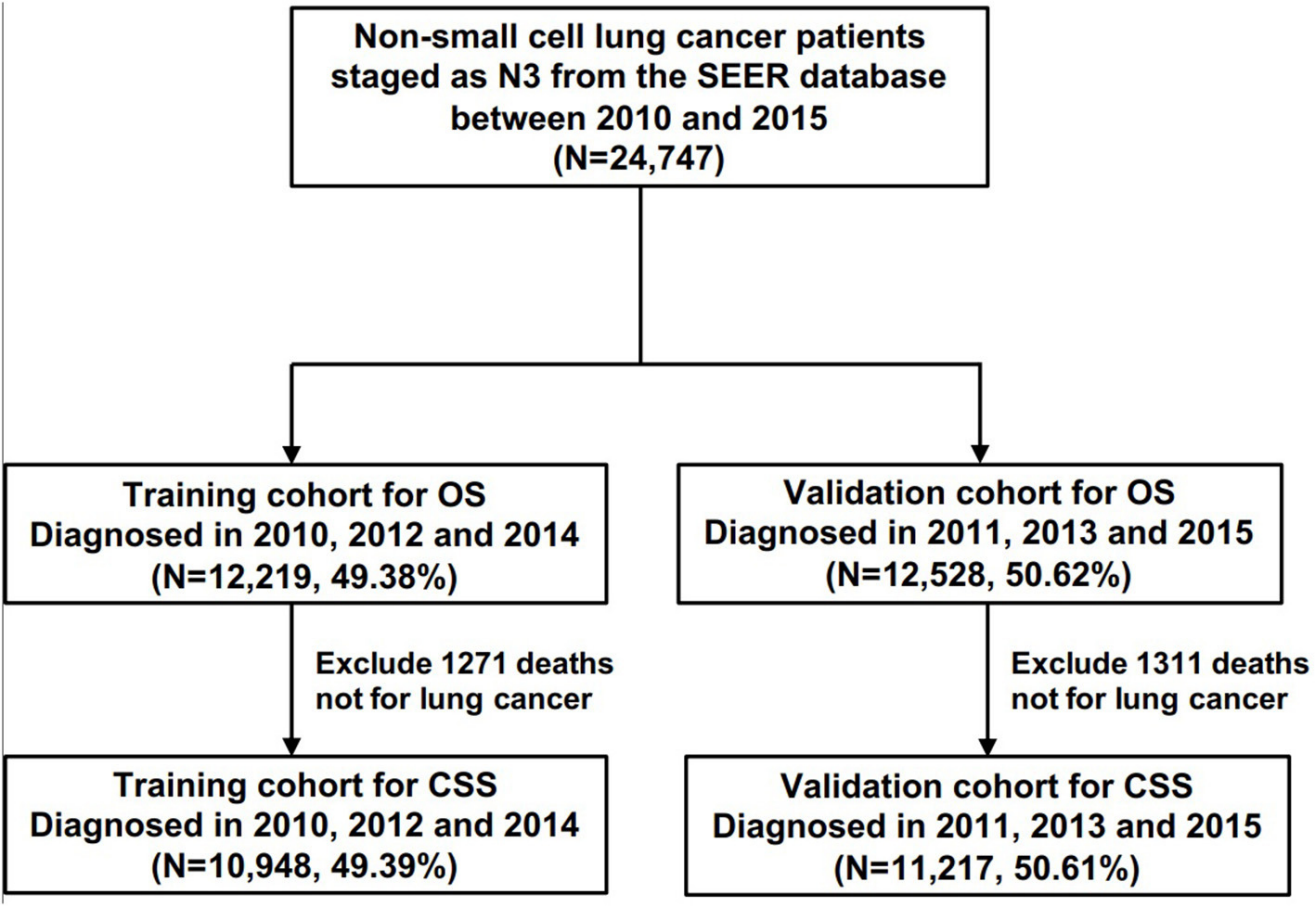
The flow chart of patients for the training and the validation cohorts for the development of predictive models.

All statistical analyses in this study were performed using R software version 3.6.3 (https://www.r-project.org) and IBM SPSS 25.0 (SPSS Inc.; Chicago, IL, USA). A two-side *p* < 0.05 was considered statistically significant. All statistical analyses were two-sided. To identify prognostic factors involved with overall survival (OS) and lung cancer-specific survival (CSS), univariate and multivariate Cox regression (LR forward) analyses were conducted among all N3-NSCLC patients. Furthermore, to develop predictive models for OS and CSS, all patients were grouped into a training cohort (2010, 2012, and 2014) and a validation cohort (2011, 2013, and 2015) in accordance with the diagnosis year (Figure 1). The distribution of the baseline characteristics between two cohorts was compared by Pearson's Chi square test. The least absolute shrinkage and selection operator (LASSO) regression analyses were performed in the training cohort to select the optimal prognostic parameters for OS and CSS, which were then used for the construction of predictive nomogram models. To evaluate the models' performance, the C-index values were calculated, which indicate discrimination ability. Additionally, using the validation cohort, calibration curves were also plotted with 500 bootstrap resamples, which demonstrate the nomogram models' agreement between the actual and the predicted survival outcome.

The survival curves were plotted using the Kaplan-Meier method and were compared by log-rank test. To further compare the survival outcome in patients receiving different treatments, propensity score matching analyses were also performed.

## Results

### Demographic Characteristics

The patients' demographic characteristics are described in Table 1. A total of 24,747 patients diagnosed with N3-NSCLC between 2010 and 2015 were finally enrolled in our study, involving 13,939 (56.3%) females and 10,808 (43.7%) males with a median age of 67 [interquartile range (IQR): 59–75] years. According to the definite records, the most common sites of distant metastasis among N3-NSCLC patients were bone (7,093, 28.7%) and lung (6,886, 27.8%), followed by brain (4,571, 18.5%) and liver (3163, 12.8%). Among them, 23.8% had more than one site of distant metastasis. Surgical resection was performed for only 505 (2.0%) patients, including sublobar resection, lobectomy, and other lung resections. As for systematic therapy, 7,129 (28.8%) patients underwent only chemotherapy, 3,035 (12.3%) patients received only radiotherapy, and 9,736 (39.3%) were recorded to receive both chemotherapy and radiotherapy.

**Table 1:** Demographic characteristics of N3-NSCLC patients.

**Characteristic**		***N* = 24,747 (%)**
Age, years	Median (IQR)	67 (59–75)
Race	White	19,010 (76.8)
	Black	3,397 (13.7)
	Others	2,340 (9.5)
Sex	Male	13,939 (56.3)
	Female	10,808 (43.7)
Diagnosis year	2010	3,847 (15.5)
	2011	3,865 (15.6)
	2012	4,070 (16.4)
	2013	4,158 (16.8)
	2014	4,302 (17.4)
	2015	4,505 (18.2)
Laterality	Right	13,145 (53.1)
	Left	10,224 (41.3)
	Unknown	1,378 (5.6)
Primary site	Upper lobe	13,041 (52.7)
	Middle lobe	991 (4.0)
	Lower lobe	5,854 (23.7)
	Main bronchus	1,237 (5.0)
	Overlapping lesion	253 (1.0)
	Unknown	3,371 (13.6)
Stage	IIIB	6,762 (27.3)
	IV	17,985 (72.7)
T stage	T1	2,767 (11.2)
	T2	5,700 (23.0)
	T3	5,930 (24.0)
	T4	8,157 (33.0)
	TX	2,193 (8.8)
Bone metastasis	Yes	7,093 (28.7)
	No	17,076 (69.0)
	Unknown	578 (2.3)
Brain metastasis	Yes	4,571 (18.5)
	No	19,561 (79.0)
	Unknown	615 (2.5)
Liver metastasis	Yes	3,163 (12.8)
	No	20,954 (84.7)
	Unknown	630 (2.5)
Lung metastasis	Yes	6,886 (27.8)
	No	17,072 (69.0)
	Unknown	789 (3.2)
Histology	ADC	13,674 (55.3)
	SCC	5,588 (22.6)
	Other NSCLC	5,485 (22.1)
Grade	I	419 (1.7)
	II	2,468 (10.0)
	III	6,389 (25.8)
	IV	347 (1.4)
	Unknown	15,124 (61.1)
Surgery	Yes	505 (2.0)
	No	24,242 (98.0)
Chemotherapy	Yes	16,865 (68.1)
	No/unknown	7,882 (31.9)
Radiotherapy	Yes	12,771 (51.6)
	No/unknown	11,976 (48.4)
Radiation sequence	After surgery	189 (0.8)
	Before surgery	46 (0.2)
	Others/unknown	270 (1.1)
	No surgery	24,242 (98.0)
Distant metastasis	Bone	2,400 (9.7)
	Brain	1,599 (6.5)
	Liver	521 (2.1)
	Lung	2,856 (11.5)
	≥two sites	5,882 (23.8)
	None	10,457 (42.3)
	Unknown	1,032 (4.2)
Systematic therapy	Chemotherapy	7,129 (28.8)
	Radiotherapy	3,035 (12.3)
	Both	9,736 (39.3)
	None	4,847 (19.6)

*NSCLC, non-small cell lung cancer; IQR, interquartile range; ADC, adenocarcinoma; SCC, squamous cell carcinoma*.

The results of univariate and multivariate Cox analyses for OS and CSS are shown in Table 2. The significant prognostic factors for OS and CSS were similar, which included age, race, diagnosis year, primary site, stage, T stage, bone, brain and liver metastases, histology, grade, surgery, and chemotherapy.

**Table 2:** Univariate and multivariate Cox regression analysis for overall survival (OS) and lung cancer-specific survival (CSS) in all patients with N3-NSCLC.

**Characteristic**		**Cox regression for OS**						**Cox regression for CSS**					
		**Univariate analysis**			**Multivariate analysis**			**Univariate analysis**			**Multivariate analysis**		
		**HR**	**95%CI**	***P*-value**	**HR**	**95%CI**	***P*-value**	**HR**	**95%CI**	***P*-value**	**HR**	**95%CI**	***P*-value**
Age	<67, median	Ref.	–	–	Ref.	–	–	Ref.	–	–	Ref.	–	–
	≥67	1.20	1.16–1.23	<0.001	1.15	1.12–1.18	<0.001	1.21	1.17–1.24	<0.001	1.16	1.13–1.19	<0.001
Race	White	Ref.	–	–	Ref.	–	–	Ref.	–	–	Ref.	–	–
	Black	0.99	0.96–1.04	0.946	0.99	0.95–1.03	0.453	0.99	0.95–1.04	0.741	0.99	0.95–1.03	0.517
	Others	0.77	0.74–0.81	<0.001	0.74	0.71–0.78	<0.001	0.77	0.73–0.81	<0.001	0.74	0.70–0.78	<0.001
Sex	Male	Ref.	–	–	Ref.	–	–	Ref.	–	–	Ref.	–	–
	Female	0.84	0.81–0.86	<0.001	0.85	0.82–0.87	<0.001	0.83	0.80–0.85	<0.001	0.84	0.82–0.87	<0.001
Diagnosis year	2010	Ref.	–	–	Ref.	–	–	Ref.	–	–	Ref.	–	–
	2011	0.94	0.90–0.98	0.005	0.96	0.92–1.00	0.065	0.93	0.88–0.97	0.002	0.94	0.90–0.99	0.017
	2012	0.99	0.95–1.04	0.636	1.00	0.96–1.05	0.936	0.97	0.92–1.02	0.191	0.98	0.93–1.03	0.334
	2013	0.95	0.91–0.99	0.020	0.96	0.92–1.00	0.075	0.92	0.87–0.96	<0.001	0.93	0.88–0.97	0.003
	2014	0.88	0.84–0.92	<0.001	0.89	0.85–0.93	<0.001	0.83	0.79–0.88	<0.001	0.84	0.80–0.88	<0.001
	2015	0.88	0.84–0.92	<0.001	0.88	0.84–0.92	<0.001	0.81	0.77–0.86	<0.001	0.81	0.77–0.85	<0.001
Laterality	Right	Ref.	–	–	Ref.	–	–	Ref.	–	–	–	–	–
	Left	1.00	0.97–1.02	0.769	0.98	0.95–1.00	0.095	1	0.97–1.03	0.85	–	–	–
	Unknown	1.17	1.10–1.24		0.92	0.85–0.98	0.015	1.15	1.08–1.23	<0.001	–	–	–
Primary site	Upper lobe	Ref.	–	–	Ref.	–	–	Ref.	–	–	Ref.	–	–
	Middle lobe	0.93	0.87–1.00	0.036	0.92	0.86–0.99	0.025	0.92	0.85–0.99	0.019	0.91	0.84–0.98	0.011
	Lower lobe	1.03	0.99–1.06	0.135	1.01	0.97–1.04	0.756	1.02	0.98–1.06	0.311	1.00	0.97–1.04	0.968
	Main bronchus	1.19	1.12–1.27	<0.001	1.13	1.07–1.21	<0.001	1.2	1.12–1.28	<0.001	1.14	1.07–1.21	<0.001
	Overlapping lesion	1.22	1.07–1.39	0.004	1.18	1.03–1.34	0.017	1.22	1.07–1.40	0.004	1.17	1.02–1.34	0.022
	Unknown	1.20	1.16–1.25	<0.001	1.09	1.04–1.15	<0.001	1.18	1.13–1.24	<0.001	1.08	1.02–1.14	0.004
Stage	IIIB	Ref.	–	–	Ref.	–	–	Ref.	–	–	Ref.	–	–
	IV	1.78	1.72–1.83	<0.001	1.53	1.47–1.58	<0.001	1.8	1.74–1.86	<0.001	1.54	1.48–1.60	<0.001
T stage	T1	Ref.	–	–	Ref.	–	–	Ref.	–	–	Ref.	–	–
	T2	1.25	1.19–1.31	<0.001	1.20	1.14–1.27	<0.001	1.27	1.20–1.34	<0.001	1.21	1.14–1.27	<0.001
	T3	1.39	1.32–1.46	<0.001	1.26	1.20–1.32	<0.001	1.41	1.34–1.49	<0.001	1.27	1.20–1.34	<0.001
	T4	1.58	1.50–1.65	<0.001	1.38	1.31–1.45	<0.001	1.61	1.53–1.69	<0.001	1.39	1.32–1.47	<0.001
	TX	1.46	1.37–1.55	<0.001	1.24	1.16–1.32	<0.001	1.47	1.37–1.57	<0.001	1.23	1.15–1.32	<0.001
Bone metastasis	Yes	1.52	1.47–1.56	<0.001	1.27	1.23–1.31	<0.001	1.53	1.48–1.58	<0.001	1.28	1.23–1.32	<0.001
	No	Ref.	–	–	Ref.	–	–	Ref.	–	–	Ref.	–	–
	Unknown	1.35	1.24–1.47	<0.001	1.06	0.93–1.20	0.409	1.35	1.23–1.48	<0.001	1.05	0.92–1.20	0.493
Brain metastasis	Yes	1.45	1.40–1.50		1.27	1.23–1.32	<0.001	1.45	1.40–1.50	<0.001	1.28	1.23–1.33	<0.001
	No	Ref.	–	–	Ref.	–	–	Ref.	–	–	Ref.	–	–
	Unknown	1.34	1.23–1.46	<0.001	0.98	0.86–1.11	0.733	1.36	1.24–1.48	<0.001	0.99	0.87–1.13	0.920
Liver metastasis	Yes	1.64	1.58–1.71	<0.001	1.30	1.25–1.36	<0.001	1.65	1.58–1.72	<0.001	1.32	1.26–1.37	<0.001
	No	Ref.	–	–	Ref.	–	–	Ref.	–	–	Ref.	–	–
	Unknown	1.27	1.17–1.38	<0.001	1.03	0.91–1.16	0.625	1.26	1.15–1.37	<0.001	1.01	0.89–1.15	0.897
Lung metastasis	Yes	1.30	1.27–1.34	<0.001	–	–	–	1.31	1.30–1.35	<0.001	–	–	–
	No	Ref.	–	–	–	–	–	Ref.	–	–	–	–	–
	Unknown	1.35	1.29–1.45	<0.001	–	–	–	1.33	1.23–1.44	<0.001	–	–	–
Histology	ADC	Ref.	–	–	Ref.	–	–	Ref.	–	–	Ref.	–	–
	SCC	1.09	1.06–1.13	<0.001	1.14	1.10–1.18	<0.001	1.1	1.06–1.14	<0.001	1.15	1.11–1.19	<0.001
	Other NSCLC	1.26	1.22–1.30	<0.001	1.13	1.09–1.17	<0.001	1.25	1.20–1.29	<0.001	1.12	1.08–1.16	<0.001
Grade	I	Ref.	–	–	Ref.	–	–	Ref.	–	–	Ref.	–	–
	II	1.23	1.09–1.38	<0.001	1.22	1.09–1.37	0.001	1.23	1.09–1.40	0.001	1.24	1.10–1.41	0.001
	III	1.49	1.33–1.66	<0.001	1.45	1.29–1.62	<0.001	1.51	1.34–1.70	<0.001	1.48	1.31–1.67	<0.001
	IV	1.69	1.45–1.96	<0.001	1.60	1.37–1.87	<0.001	1.66	1.41–1.96	<0.001	1.61	1.37–1.91	<0.001
	Unknown	1.42	1.28–1.59	<0.001	1.33	1.19–1.48	<0.001	1.42	1.26–1.60	<0.001	1.35	1.20–1.52	<0.001
Surgery	Yes	0.63	0.57–0.70	<0.001	0.71	0.64–0.79	<0.001	0.64	0.58–0.71	<0.001	0.73	0.65–0.81	<0.001
	No	Ref.	–	–	Ref.	–	–	Ref.	–	–	Ref.	–	–
Chemotherapy	Yes	0.43	0.41–0.44	<0.001	0.42	0.41–0.44	<0.001	0.42	0.41–0.43	<0.001	0.41	0.40–0.43	<0.001
	No/unknown	Ref.	–	–	Ref.	–	–	Ref.	–	–	Ref.	–	–
Radiotherapy	Yes	0.84	0.82–0.86	<0.001	–	–	–	0.85	0.82–0.87	<0.001	–	–	–
	No/unknown	Ref.	–	–	–	–	–	Ref.	–	–	–	–	–
Radiation sequence	After surgery	Ref.	–	–	–	–	–	Ref.	–	–	–	–	–
	Before surgery	0.63	0.42–0.92	0.017	–	–	–	0.64	0.42–0.96	<0.001	–	–	–
	Others/unknown	0.96	0.78–1.19	0.699	–	–	–	0.99	0.79–1.25	0.957	–	–	–
	No surgery	1.47	1.25–1.73	<0.001	–	–	–	1.49	1.25–1.77	<0.001	–	–	–

### Predictive Nomogram

**Figure 2:**
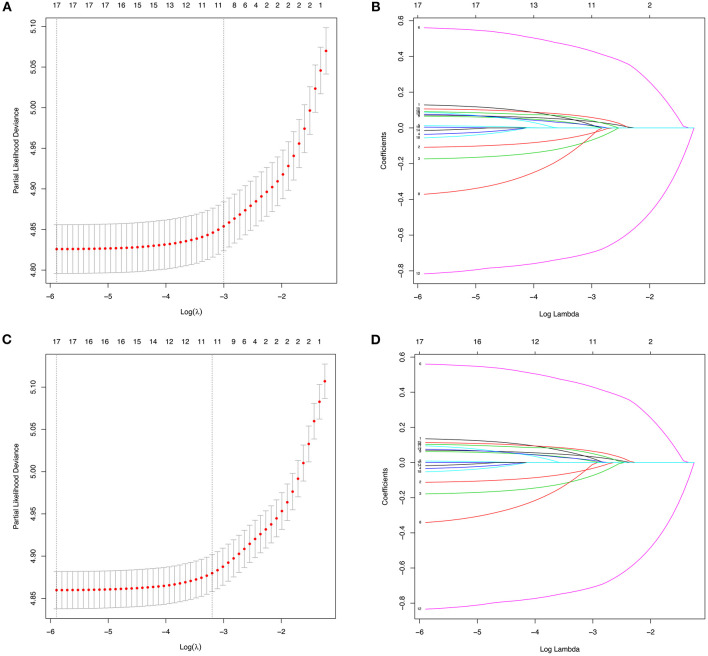
The identification of predictive prognostic factors for overall survival (OS) **(A,B)** and lung cancer-specific survival (CSS) **(C,D)** in the training cohorts of patients with N3-NSCLC by the least absolute shrinkage and selection operator (LASSO) regression.

**Figure 3:**
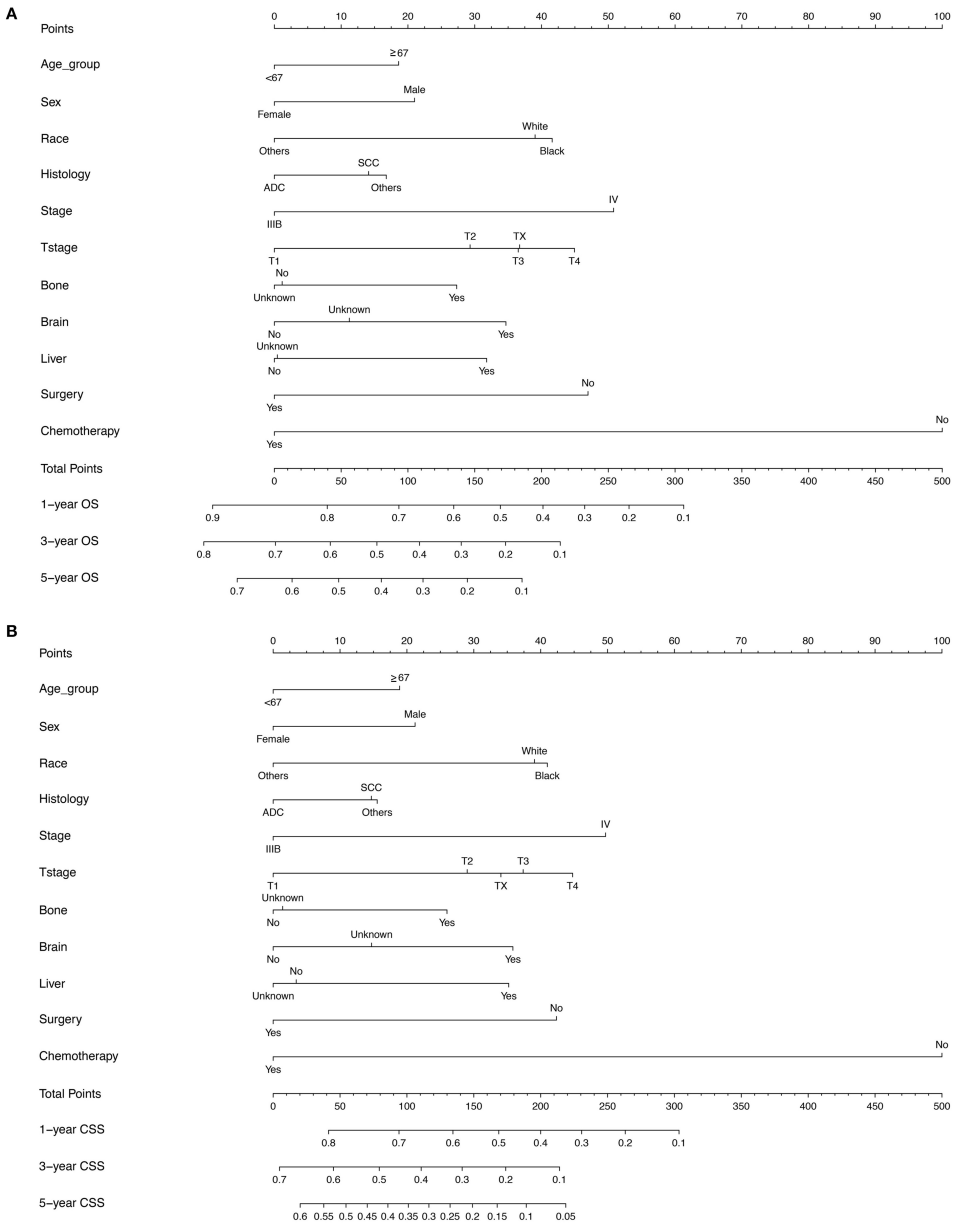
The nomogram models for predicting **(A)** OS **(B)** lung CSS in patients with N3-NSCLC. ADC, adenocarcinoma; SCC, squamous cell carcinoma.

According to the diagnosis year, all patients were divided into the training cohort (OS: *N* = 12,219, 49.38%; CSS: *N* = 10,948, 49.39%) and the validation cohort (OS: *N* = 12,528, 50.62%; CSS: *N* = 11,217, 50.61%; Figure 1). The comparison of patients' baseline characteristics between two cohorts for OS are shown in Table 3, most of which demonstrates similar distribution. Prior to the construction of nomogram models, the training cohorts were used to select prognostic parameters by LASSO regression (Figure 2). As shown in Figure 2, the training cohorts for OS (Figures 2A,B) and CSS (Figures 2C,D) also demonstrated the similar results, both of which identified 11 prognostic parameters with one-fold standard error of minimum criterion. These parameters were as follows: age, sex, race, histology, stage, T stage, bone, brain and liver metastases, surgery, and chemotherapy. Then the nomogram models for OS (Figure 3A) and CSS (Figure 3B) were developed.

**Table 3:** Baseline characteristics in the training and the validation cohorts of N3-NSCLC patients.

**Characteristic**		**Training cohort** **(*N* = 12,219)**	**Validation cohort** **(*N* = 12,528)**	***P*-value**
Age	<67, median	6,059 (49.6)	6,183 (49.4)	0.723
	≥67	6,160 (50.4)	6,345 (50.6)	
Race	White	9,396 (76.9)	9,614 (76.7)	0.901
	Black	1,665 (13.6)	1,732 (13.8)	
	Others	1,158 (9.5)	1,182 (9.4)	
Sex	Male	6,905 (56.5)	7,034 (56.1)	0.572
	Female	5,314 (43.5)	5,494 (43.9)	
Laterality	Right	6,519 (53.4)	6,626 (52.9)	0.027
	Left	4,978 (40.7)	5,246 (41.9)	
	Unknown	722 (5.9)	656 (5.2)	
Primary site	Upper lobe	6,449 (52.8)	6,592 (52.6)	0.429
	Middle lobe	494 (4.0)	497 (4.0)	
	Lower lobe	2,833 (23.2)	3,021 (24.1)	
	Main bronchus	610 (5.0)	627 (5.0)	
	Overlapping lesion	123 (1.0)	130 (1.0)	
	Unknown	1,710 (14.0)	1,661 (13.3)	
Stage	IIIB	3,370 (27.6)	3,392 (27.1)	0.381
	IV	8,849 (72.4)	9,136 (72.9)	
T stage	T1	1,388 (11.4)	1,379 (11.0)	0.001
	T2	2,783 (22.8)	2,917 (23.3)	
	T3	2,847 (23.3)	3,083 (24.6)	
	T4	4,036 (33.0)	4,121 (32.9)	
	TX	1,165 (9.5)	1,028 (8.2)	
Bone metastasis	No	8,492 (69.5)	8,584 (68.5)	0.072
	Yes	3,428 (28.1)	3,665 (29.3)	
	Unknown	299 (2.4)	279 (2.2)	
Brain metastasis	No	9,641 (78.9)	9,920 (79.2)	0.138
	Yes	2,250 (18.4)	2,321 (18.5)	
	Unknown	328 (2.7)	287 (2.3)	
Liver metastasis	No	10,344 (84.7)	10,610 (84.7)	0.055
	Yes	1,536 (12.6)	1,627 (13.0)	
	Unknown	339 (2.8)	291 (2.3)	
Lung metastasis	No	8,446 (69.1)	8,626 (68.9)	0.005
	Yes	3342 (27.4)	3,544 (28.3)	
	Unknown	431 (3.5)	358 (2.9)	
Histology	ADC	6,601 (54.0)	7,073 (56.5)	<0.001
	SCC	2,807 (23.0)	2,781 (22.2)	
	Other NSCLC	2,811 (23.0)	2,674 (21.3)	
Grade	I	202 (1.7)	217 (1.7)	0.217
	II	1,194 (9.8)	1,274 (10.2)	
	III	3,233 (26.5)	3,156 (25.2)	
	IV	173 (1.4)	174 (1.4)	
	Unknown	7,417 (60.7)	7,707 (61.5)	
Surgery	Yes	11,983 (98.1)	12,259 (97.9)	0.248
	No	236 (1.9)	269 (2.1)	
Chemotherapy	Yes	3,924 (32.1)	3,958 (31.6)	0.387
	No/unknown	8,295 (67.9)	8,570 (68.4)	
Radiotherapy	Yes	5,903 (48.3)	6,073 (48.5)	0.804
	No/unknown	6,316 (51.7)	6,455 (51.5)	

*NSCLC, non-small cell lung cancer; ADC, adenocarcinoma; SCC, squamous cell carcinoma*.

**Figure 4:**
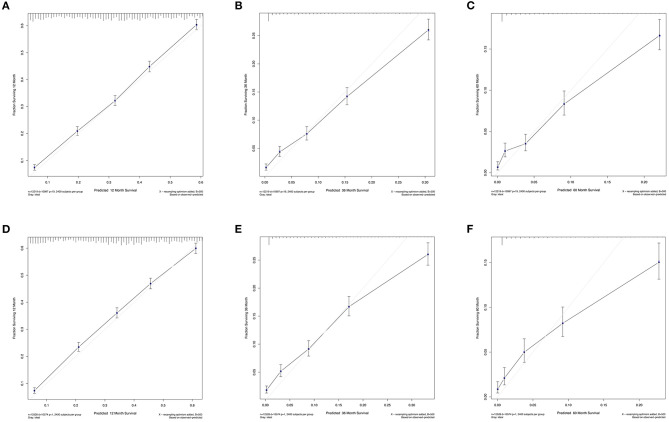
Calibration curves for the nomogram model of predicting 1-year **(A,D)**, 3-year **(B,E)**, and 5-year **(C,F)** OS in patients with N3-NSCLC. **(A–C)** Internal validation using the training cohort. **(D–F)** External validation using the validation cohort.

**Figure 5:**
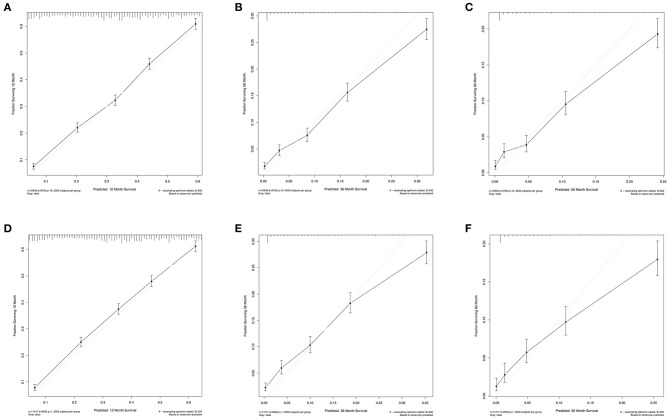
Calibration curves for the nomogram model of predicting 1-year **(A,D)**, 3-year **(B,E)**, and 5-year **(C,F)** Lung CSS in patients with N3-NSCLC. **(A–C)** Internal validation using the training cohort. **(D–F)** External validation using the validation cohort.

The C-index values were calculated to assess predictive performance in both training and validation cohorts. In the training cohorts, the two nomogram models presented much the same index, which was 0.696 (95%CI: 0.684–0.708, *P* < 0.001). And in the validation cohorts, similar values of C-index were also observed for OS (0.698, 95%CI: 0.686–0.710, *P* < 0.001) and CSS (0.699, 95%CI: 0.687–0.711, *P* < 0.001). Besides, calibration analyses for the OS (Figure 4) and CSS (Figure 5) nomogram models were performed to evaluate the agreement between the predicted and the actual survival. The results of internal (Figures 4A–C, 5A–C) and external validation (Figures 4D–F, 5D–F) were plotted using the training and validation cohorts, respectively. For both models, the calibration curves of 1-year OS (Figures 4A,D) and CSS (Figures 5A,D) showed perfect overlapping between the ideal line and the true line, while the curves of 3-year (Figures 4B,E, 5B,E) and 5-year (Figures 4C,F, 5C,F) also demonstrated great agreement performance, though they were not comparable to those of 1-year.

### Survival Analysis

**Figure 6:**
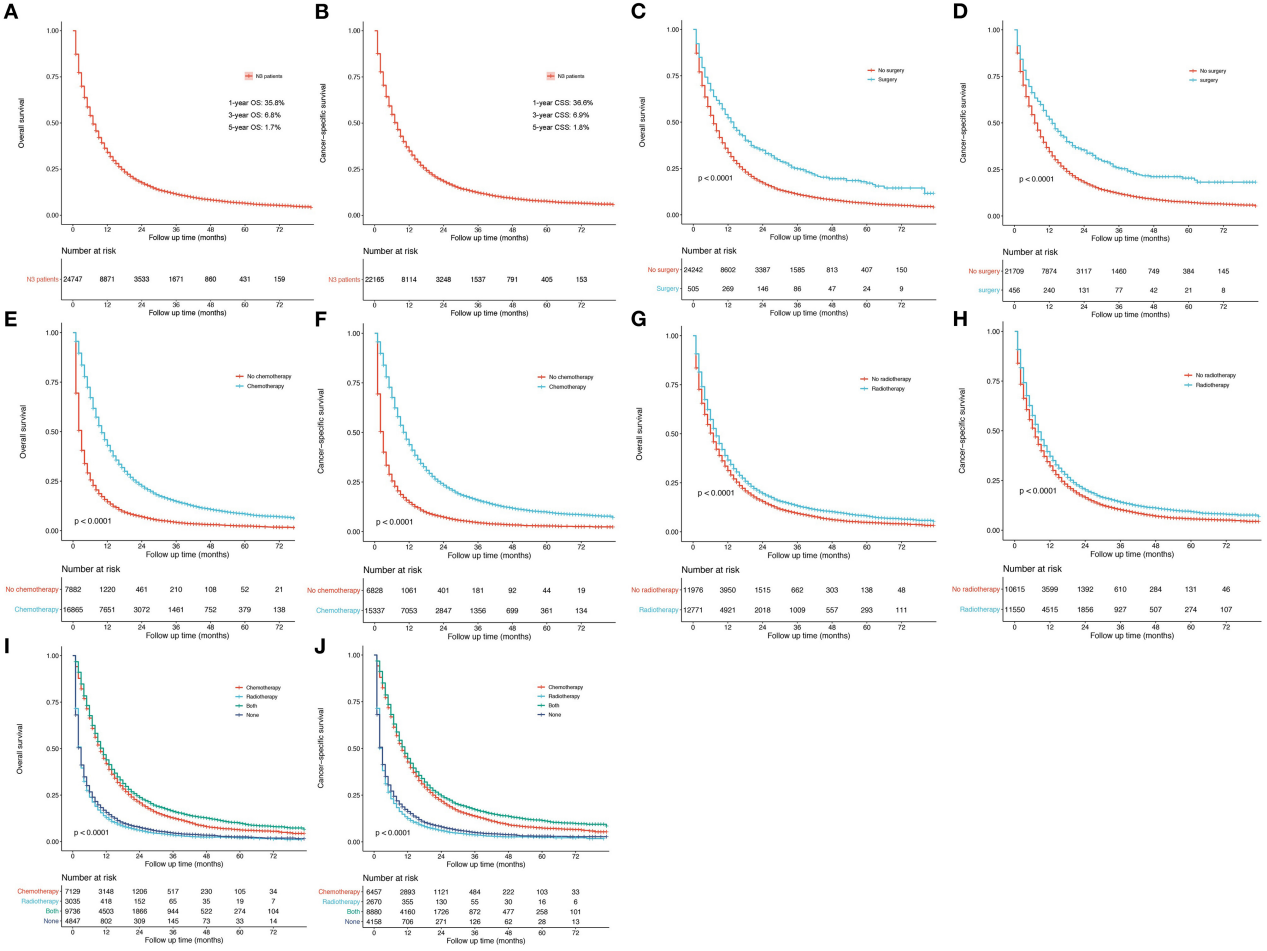
Survival curves. **(A,B)** OS and lung cancer-specific OS for all N3-NSCLC patients. **(C,D)** Surgery vs. no surgery. **(E,F)** Chemotherapy vs. no chemotherapy. **(G,H)** Radiotherapy vs. no radiotherapy. **(I,J)** Groups distributed by systematic therapy (only chemotherapy, only radiotherapy, both, and none).

A relatively poor survival was observed among N3-NSCLC patients. The 1-, 3-, and 5-year OS rates were 35.8, 6.8, and 1.7% (Figure 6A), respectively, while the corresponding CSS rates was 36.6, 6.9, and 1.8% (Figure 6B), respectively. Patients who underwent surgery (Figures 6C,D), chemotherapy (Figures 6E,F), or radiotherapy (Figures 6G,H) demonstrated significantly better OS and CSS than those who did not (*P* < 0.001). It was observed that chemotherapy or chemotherapy plus radiotherapy could be more beneficial to the survival outcome of N3-NSCLC patients than those only receiving radiotherapy or none of treatments (Figures 6I,J).

**Figure 7:**
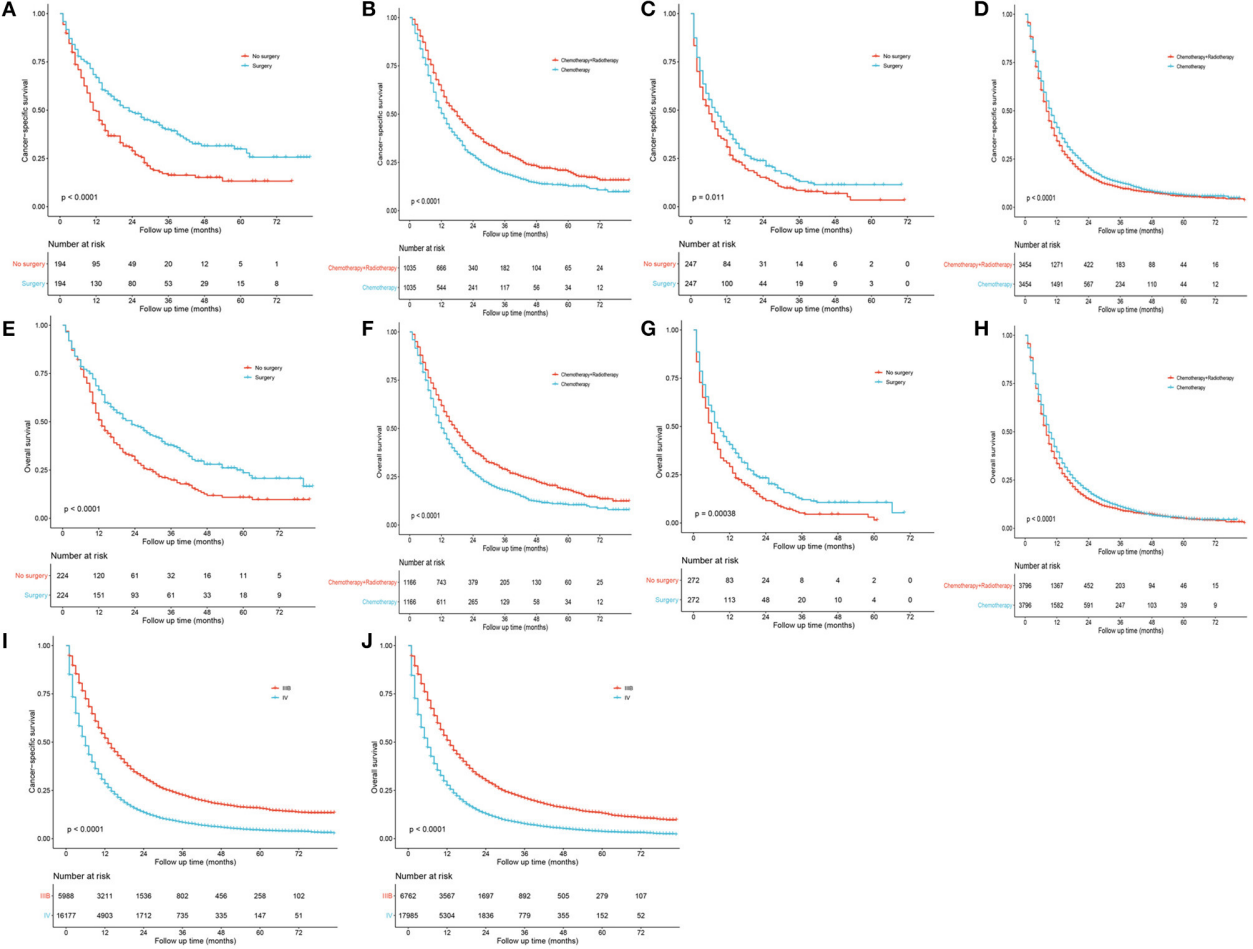
Survival curves after propensity score matching. **(A,E)** Surgery vs. no surgery for IIIB patients. **(B,F)** Chemotherapy vs. chemotherapy plus radiotherapy for IIIB patients. **(C,G)** Surgery vs. no surgery for IV patients. **(D,H)** Chemotherapy vs. chemotherapy plus radiotherapy for IV patients. **(I,J)** Stage IIIB vs. IV.

Considering the possible bias of treatment groups, propensity score matching (PSM) analyses were used. Using the nearest neighbor matching algorithm, baseline characteristics in different treatment groups were adjusted for balance in patients staged as IIB and IV, respectively. After matching with a ratio of 1:1 (caliper value = 0.01), patients undergoing surgery showed significantly better OS and CSS than those who did not for both stages IIIB (Figures 7A,E; *P* < 0.001) and IV patients (Figure 7C, *P* = 0.011 and Figure 7G, *P* < 0.001). To further evaluate the effects of radiotherapy on survival of N3-NSCLC, the baseline was well-matched with a ratio of 1:1 (caliper value = 0.001) between patients undergoing chemotherapy and chemotherapy plus radiotherapy. For IIIB patients, chemotherapy plus radiation demonstrated significantly better survival than only chemotherapy (Figures 7B,F; *P* < 0.001). However, it seemed that the additional radiotherapy did not increase survival benefits to IV-N3 patients receiving chemotherapy (Figures 7D,H).

## Discussion

Generally, N3-positive is regarded as the terminal stage of NSCLC, and these patients have few opportunities receiving radical resection of tumors. N3 disease was defined as the involvement of one of scalene zone, supraclavicular, or contralateral hilar/mediastinal lymph nodes, which has been applied since 1986 for staging NSCLC (9). However, circumscribed advances have been made in the past decades for the clinical management of N3-positive NSCLC patients. The current commonly recommended and accepted therapy is concurrent chemoradiotherapy (1, 2, 4–7). Because of the rapid development of gene detection technologies, the concept of precision medicine was proposed, and the target therapy is the most potential for curing cancer. In recent years, the immunotherapy has also obtained encouraging curative effects. Novel multiple modalities are required for N3-NSCLC. Hence, conducting the prognostic study or developing a prognostic model among N3-NSCLC patients may provide helpful indications.

N3-positive NSCLC will be staged as IIIB if there is no evidence for any distant metastasis, and those with distant metastasis will be assigned into the later IV stage. Additionally, variations of involved lymph nodes also contributed to the heterogeneity of this population. Most of previous studies and clinical trials were conducted with a limited number of N3-NSCLC patients, even excluding those with positive supraclavicular nodes (4–7). In these studies, there was lack of subgroup analyses for the N3 disease and whether their conclusions for IIIB patients were suitable for N3-positive patients remains debated.

In this prognostic analysis, we first developed predictive models for survival among N3-NSCLC patients, and compared the clinical outcome in different subgroups based on the large data from the SEER program. Given that very few studies reported the survival related to N3 disease, we aim to propose some retrospective evidences for helping designing future studies to improve therapy. The lung cancer staging project by International Association for the Study of Lung Cancer (IASLC) in 2015 enrolled 17,477 NSCLC patients worldwide, among which the incidence rate of N3 was 8.86% (1,548/17,477) (3). Most of them (909/1,548, 58.72%) were staged as T4, and 408 (26.36%) cases were proved to have distant metastasis. In our study, a total of 24,747 N3-NSCLC patients diagnosed between 2010 and 2015 were retrospectively analyzed, including 2,767 (11.2%) cases of T1, 5,700 (23.0%) of T2, 5,930 (24.0%) of T3, and 8,157 (33.0%) of T4. The definite records showed that 10,858 (64.08%) patients had distant metastasis.

The 5-year OS rates for patients staged as IIIB based on the 7th and 8th edition TNM staging system were 19 and 26%, respectively (3). Since such data of survival for the population with N3 disease was rarely reported, our analysis may provide references. Of all patients we included, the 1-, 3-, and 5-year OS rates were 35.8, 6.8, and 1.7%, respectively. Specifically, the corresponding rates were 52.8, 13.2, and 4.1% for IIIB-N3, and 29.5, 4.3, and 8.5% for IV-N3. It could be inferred N3-IIIB patients had a poorer long-term survival than all IIIB individuals. This study hypothesized that the previous viewpoints of therapeutic strategy for N3-NSCLC could be optimized in detail.

Apart from the conventional factors, the prognostic analysis indicated several parameters might have a significant effect on survival (Table 2). Patients of American Indian/AK Native and Asian/Pacific Islander had a better prognosis compared with white or black patients. In addition, females demonstrated a greater survival outcome than males, which might be attributed to the larger proportion of smoker in males, though such information of smoking history was available in the SEER database. Furthermore, we also observed that receiving surgery, radiotherapy, and chemotherapy could offer more survival benefits to N3 patients. To quantificationally identify the parameters' contributions to the survival, we first developed predictive nomogram models for OS and CSS (Figure 3). Clinicians and patients worldwide can easily use the nomograms to calculate the survival rates of N3-NSCLC patients. Our models demonstrated great predictive performance with high C-index values. Besides, calibration curves of both internal and external validation also showed powerful agreement between the predicted and the actual survival outcome, especially for 1-year OS and CSS, which were the most concerned (Figures 4, 5).

To compare the survival outcome in different treatment groups, survival curves were plotted with similar results as Cox regression analysis (Figure 6). IIIB-NSCLC is usually regarded as inoperable. However, surgical resection might increase more survival benefits when combining with induction therapy (10–15). Recently, Raman et al. reported 935 matched patient pairs by PSM and found that surgical resection for N3-NSCLC could improve long-term survival with chemoradiation (8), which was consistent with our study (IIIB: Figures 7A,E; IV: Figures 7C,G,I,J). Besides, in Figures 6I,J, the survival curves of patients receiving chemotherapy with and without radiotherapy are very close. Considering the data bias, baseline characteristics were matched by PSM between the chemotherapy-only group and the chemotherapy plus radiotherapy group. Among IIIB patients, chemotherapy plus radiation showed significantly better survival than only chemotherapy (Figures 7B,F). However, for stage IV patients receiving chemotherapy, radiotherapy did not increase additional survival benefits (Figures 7D,H). Those undergoing radiation seemed to have slightly worse survival over the short term.

There were several limitations in this study that required declaration. First, as a retrospective cohort from the SEER database, some inherent data bias could not be totally eliminated, though there were a large number of N3-NSCLC patients. The use of PSM may be able to decrease the selection bias between different treatment groups. Second, some details of patients' characteristics are unavailable in the SEER database, such as smoking history, regimens of chemotherapy and radiotherapy, and time of therapy. Especially, enrolled N3 patients in our study were staged according to TNM Staging System of AJCC 7th edition because of the update lag in the SEER database. Thus, these N3 patients can only be divided into IIIB-N3 and IV-N3, which does not include IIIC-N3 as in the TNM staging system of AJCC 8th edition. In spite of these, our predictive models performed very well in the aspects of discrimination and agreement, which can be of clinical significance. Finally, the multimodality for N3-NSCLC patients was limited by the lack of records in the SEER program. The novel encouraging treatments such as target therapy and immunotherapy would play a critical role in the management of terminal-stage NSCLC patients including N3 disease.

## Conclusions

This study reported a prognostic study for N3-NSCLC patients and first developed predictive nomogram models for survival outcome. The models performed very well and can be used by clinicians and patients worldwide. Furthermore, the survival analyses by PSM indicated that patients undergoing surgery might have better long-term survival despite locally-advanced or metastatic disease. Compared with those receiving only chemotherapy, the extra radiation therapy could significantly improve survival among IIIB patients. However, for IV patients, radiotherapy had little benefits to chemotherapy treatments alone in terms of survival.

## Data Citation

[SEER] 2010-2015; the Surveillance, Epidemiology, and End Results database; (http://seer.cancer.gov/).

## Data Availability Statement

The original contributions presented in the study are included in the article/supplementary material, further inquiries can be directed to the corresponding author/s.

## Ethics Statement

Ethical review and approval was not required for the study on human participants in accordance with the local legislation and institutional requirements. The patients/participants provided their written informed consent to participate in this study.

## Author Contributions

YW and CH: conception and design and provision of study materials or patients. CH and ZL: collection and assembly of data. XS, ZL, and FZ: data analysis and interpretation. All authors: manuscript writing and final approval of manuscript.

## Conflict of Interest

The authors declare that the research was conducted in the absence of any commercial or financial relationships that could be construed as a potential conflict of interest.
